# Irrational prescription of surfactant replacement therapy in neonatal respiratory distress

**DOI:** 10.1371/journal.pone.0268774

**Published:** 2022-06-16

**Authors:** Nader Jahanmehr, Reyhane Izadi, Abbas Habibolahi, Setareh Yousefzadeh, Soheila Khodakarim

**Affiliations:** 1 Health Economics, Management and Policy Department, Virtual School of Medical Education & Management, Shahid Beheshti University of Medical Sciences, Tehran, Iran; 2 Prevention of Cardiovascular Disease Research Center, Shahid Beheshti University of Medical Sciences, Tehran, Iran; 3 Neonatal Health Department, Population, Family and School Health Office, Deputy of Health, Ministry of Health and Medical Education, Tehran, Iran; 4 Social Determinants of Health Research Center, Health Research Institute, Babol University of Medical Sciences, Babol, Iran; 5 Department of Biostatistics, School of Medicine, Shiraz University of Medical Sciences, Shiraz, Iran; The University of Mississippi Medical Center, UNITED STATES

## Abstract

**Purpose:**

Respiratory distress is known as one of the leading causes of neonatal death. In recent decades, surfactant therapy has revolutionized respiratory failure. Since the implementation of the health system reform plan as well as the allocation of new financial resources for health system in Iran, the rate of irrational prescription has increased and prescription of surfactant for neonates, has raised unexpectedly, which is thought to be due to irrational prescriptions partly. The present study aimed to determine the rate of irrational prescription of surfactant for neonates with respiratory distress.

**Methods:**

This research was a cross-sectional descriptive study, which was conducted retrospectively. In the current study, determining the rate of irrational prescription was done in terms of the surfactant prescription guideline. Finally, the medical data of 846 neonates who underwent surfactant therapy in Iran in 2018, were extracted from the information system of the Ministry of Health and the neonatal medical records.

**Results:**

The results show that drug selection index, dose index, and time index were irrational for 14.30%, 27.42%, and 1.06% of neonates, respectively. Moreover, the total index of drug prescription, which is a combination of the above-mentioned three components, was found to be irrational for 31.47% of neonates.

**Conclusions:**

The results of the present study are considered as a warning to providers and decision makers in the field of neonatal health to reduce irrational prescriptions of surfactant for neonates. This study suggests the use of educational interventions in order to reduce irrational prescriptions due to drug selection as well as the use of computer alert approaches in order to reduce irrational prescriptions due to wrong dose.

## 1 Introduction

Respiratory distress diseases are one of the leading causes of neonatal death in developing countries [1]. Moreover, they are known as one of the most important causes of neonatal intensive care unit (NICU)admission. Transient tachypnea, newborn respiratory distress syndrome (NRDS), pneumonia, and meconium aspiration syndrome are common diseases of respiratory distress among neonates, of which NRDS is considered as the most common one [2].

NRDS, formerly known as hyaline membrane disease, is a pulmonary disorder caused by the alveolar surfactant deficiency. Correspondingly, this deficiency reduces the surface tension of the alveoli and consequently leads to micro-atelectasis and the reduced lung volume [3].

NRDS is often resulted from a failure in the production of pulmonary surfactant, structural lung impairment, neonatal infections [4, 5], or genetic problems related to surfactant proteins [5]. Moreover, male sex, mother with a history of diabetes, and white skin are known as the risk factors for this disease. As well, its incidence decreases with gestational age increasing, from 86% at 24 weeks to less than 1% at 39 weeks [6, 7].

NRDS manifests shortly after birth with some symptoms such as rapid heartbeat, pectus excavatum, moaning, blue skin, and oral mucosa [8], and it may possibly lead to the development of acute complications such as pneumothorax and pulmonary interstitial emphysema, Necrotizing enterocolitis, jaundice, and anemia. Necrotizing enterocolitis, jaundice, and anemia are the other complications caused by this disease [7].

The results of a previous study in Iran showed that 65.6% of neonates under 34 weeks of gestation age had this disease [9]. A Japanese scientist named Fujiwara has conducted a significant study in 1980 and for the first time, treated ten premature neonates with NRDS using an artificial surfactant [10].

In recent decades, surfactant therapy has revolutionized the treatment of NRDS. Nowadays, there are a variety of artificial and natural surfactants in the health market worldwide [11], but the evidence showed the superiority of its natural varieties [12]. Fortunately, four natural surfactants exist in the Iranian health market, including alveofact, survanta, BLES (Bovine lipid extract surfactant), and Cursorf [13, 14].

According to a previously conducted interview with the directors of the Neonatal Health Department of the Ministry of Health, and based on the unpublished information of the above-mentioned department, the surfactant prescription among Iranian newborns has been rapidly growing in recent years. According to policy makers in the field of neonatal health in Iran, it was thought that the increasing trend in surfactant prescription might be due to irrational prescriptions. As defined by World Health Organization (WHO), rational prescriptions refer to prescriptions in which a patient receives the right drug at the right dose and right time, otherwise it is considered as irrational prescriptions [15]. In this regard, the absence of drug prescription rules, the existence of soon-to-be-expired and expired drugs, and lack of medical guidelines are the factors leading to irrational prescription [16]. Accordingly, irrational prescription threatens patient’s health status by causing some possible side effects [17], challenging health equity, imposing a lot of financial pressure on insurance, and ultimately by leading to inadequate allocation of resources and the reduced quality of health services [18]. Numerous international studies have previously reported irrational prescriptions for cesarean section [19], medication, and neonatal health services [19, 20].

Although the irrational prescription of many drugs has already been identified, the irrational prescription of surfactant has not yet been investigated. Therefore, the present study was conducted at the request of the Ministry of Health of Iran with the aim of determining the rate of irrational prescriptions of Surfactant in the treatment of neonatal respiratory distress.

## 2 Methods

This research has an ethical statement from the National Ethics Committee in Iranian Biomedical Research, which was registered with the code of ethics approval “IR.TUMS.MEDICINE.REC.1399.516” on 2020-09-22, and all the methods adopted in the present study were in accordance with the relevant instructions and regulations. According to the approval of the Ethics Committee, in this study, there was no need to obtain any type of consent from the participants (written or oral).

### 2.1 Data collection instrument

This research was a cross-sectional descriptive study that was performed retrospectively. The national guideline for surfactant prescription in neonates with respiratory distress was the instrument used in the present study in order to determine the rate of irrational prescriptions. Thereafter, the required data were collected using the information system of the Iranian Maternal and Neonatal Network (IMAN Net) as well as the neonatal medical records. Based on this guideline and the WHO definition of rational prescription, each prescription was evaluated using the following three filters: right drug, right dose, and right time, respectively.

#### 2.1.1 Drug selection index (filter1)

The above-mentioned guideline recommends four indicators for the prescription of surfactant in neonates with respiratory distress. Of note, no indicator is superior to another one. The status of all these four indicators and their constituting variables were determined for each one of the cases of surfactant prescription. Afterward, each indicator and each one of its variables were placed in only one of the following three situations: rational (compliant with the guideline), irrational (non-compliant with the guideline), or unverifiable (missing data).

An indicator was considered to be rational when both of its constituting variables were rational, and if only one irrational variable existed (regardless of the status of another variable), that indicator was considered irrational for that prescription. Similarly, an indicator for a prescription was considered to be unverifiable either when both variables were unverifiable or when one variable was unverifiable and another variable was rational.

Next, simultaneous evaluation of all these four indicators revealed those patients for whom drug selection was irrational. The drug selection index was considered irrational when all these four indicators emphasized on the intended prescription being irrational. In addition, it was considered rational when at least one indicator confirmed its rationality. In other cases, which were a combination of irrational and unverifiable indicators, the drug selection index was considered as unverifiable.

The definitions of the surfactant prescription indicators for neonates based on the national guideline in Iran are as follows:

Indicator 1: Premature neonates who need endotracheal intubation during postpartum resuscitation in the delivery / operating room.

Indicator 2: Premature neonates reaching the stabilized health status in the delivery / operating room with nasal continuous positive airway pressure (NCPAP) and also need to increase continuous positive airway pressure (CPAP) to a maximum of 8 cm/H_2_O and FIO_2_ to more than 30% to 40% in order to maintain arterial oxygen saturation within an acceptable range.

Indicator 3: Premature neonates showing typical respiratory distress syndrome (RDS)radiographic symptoms in the first 48 hours of life with a chest radiograph.

Indicator 4: Premature or mature neonates with respiratory diseases who require endotracheal intubation.

Considering the importance and sensitivity of the analysis of drug selection index, besides the national guideline (Instrument 1) described earlier, in the present study, an international guide (instrument 2 (Clinical guideline of Surfactant Administration in the NICU in the Royal children’s hospital of Melbourne)) used in Australia previously, was used to determine the rate of irrational surfactant prescriptions. Moreover, two criteria were considered for selecting the second guideline. First, it should be approved by the specialized research team; and second, it should maximize the verification based on the available data.

#### 2.1.2 Dose index (filter2)

In this step, based on the guideline, a "standard dose" (according to the standard dose range for curosurf) was determined for each neonate in terms of the neonate’s weight and the type of prescribed surfactant. Afterward, the prescribed dose was compared with the standard dose, and if they were completely compliant, the dose index was considered rational, and if data were missing, the dose index was considered unverifiable.

Finally, if the prescribed dose was lesser or more than the standard dose, at the discretion of the research team, the deviation was ignored up to a maximum of ±0.50 for three surfactants, including Alveofact, BLES, and Survanta. Additionally, up to a maximum of 0.09 for Curosurf, these doses were considered as rational. Moreover, these are reported in the findings under the name of rational doses without any sensitivity. However, if the prescribed dose, which was less or more than the standard dose, was more than the above figure, we considered the dose index as irrational.

#### 2.1.3 Time index (filter3)

According to the standard protocol, if the surfactant is prescribed up to 48 hours after birth, it is right-time and rational, but if the prescription occurs by passing 48 hours from birth, it is wrong-time and irrational. Finally, in the case of missing data, this index was considered to be unverifiable.

#### 2.1.4 Total Index of Drug Prescribing (TIDP)

TIDP refers to a combination of the above-mentioned three indexes. Correspondingly, each prescription is evaluated using the following three filters: right drug, right dose, and right time, respectively. In the current study, first, the number of patients who were not clinically eligible for receiving surfactant and for whom the drug selection index was irrational was determined. Subsequently, we determined the number of patients who had the clinical condition to receive surfactant, but they had received it at the wrong dose or the wrong time. Finally, we determined the number of patients for whom the proper verification was impossible due to missing data. It is noteworthy that TIDP consists of three indexes (including the index of rational prescribing (IRP), the index of irrational prescribing (IIP), and the index of unverifiable prescribing (IUNVERFP)). In fact, the sum of IRP and IIP is as same as the index of verifiable prescribing (IVERFP).

- TIDP = IVERFP + IUNVERFP = IRP + IIP + IUNVERFP
- IVERFP = IRP + IIP
- IRP = Rational Drug and Rational Dose and Rational Time
- IIP = Irrational Drug OR Rational Drug but Irrational Dose OR Rational Drug and Rational Dose but Irrational Time OR Rational Drug but Irrational of Dose and Time OR Irrational of Drug and Dose and Time
- IUNVERFP = unverifiable Drug OR verifiable Drug but unverifiable Dose OR verifiable Drug and verifiable Dose but unverifiable Time OR verifiable Drug but unverifiable of Dose and Time OR unverifiable of Drug and Dose and Time

### 2.2 Population and sampling

The study population included all neonates who underwent surfactant therapy in Iran in 2018. In the present study Multi-stage cluster-stratified sampling was used as the sampling method. Firstly, all provinces of Iran were classified based on the national five divisions. Thereafter, from each region, the province with the highest number of surfactant prescriptions was selected and from that province, the hospitals with the highest number of surfactant prescriptions were selected. Finally, the sample was selected using simple random sampling based on the list of surfactant-treated neonates in each hospital. Each hospital’s officials were then asked to scan the medical records of the selected neonates and to send them to the IMAN Net. In the current study, considering p = 0.5, α = 0.05, β = 0.20, and d = 0.05, the sample size was estimated as 784 surfactant prescriptions. However, a total of 846 prescriptions were investigated according to the proportion of the number of the samples specified for each hospital.

### 2.3 Data analysis

Data analysis was performed using Stata ver. 16 (Stata Corp, College Station, Texas 77845 USA). Mean and standard deviation were used to report quantitative variables and frequency and percentage were used to report qualitative variables as well.

## 3 Results

**Fig 1:**
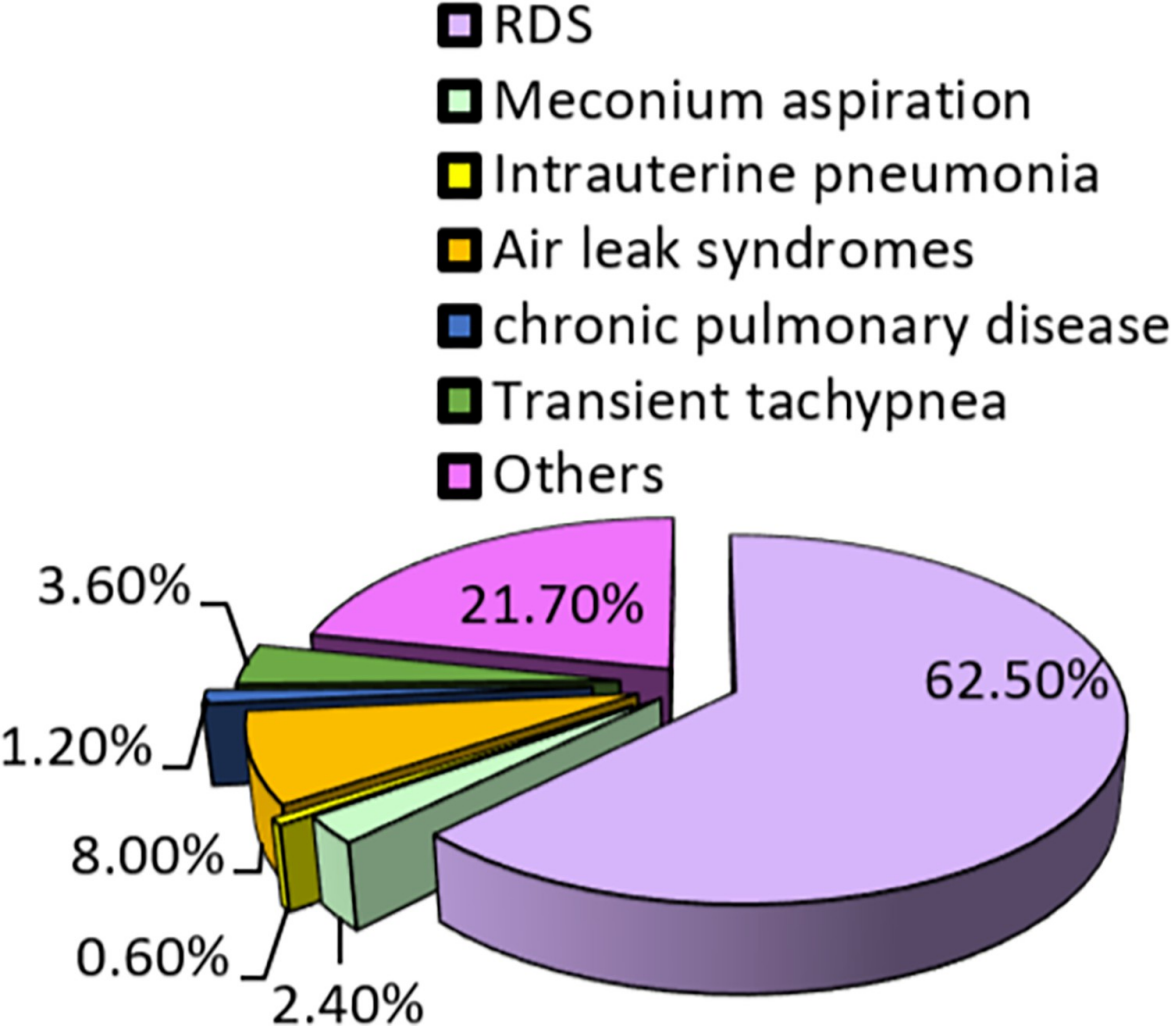
Neonates by diagnosis.

**Fig 2:**
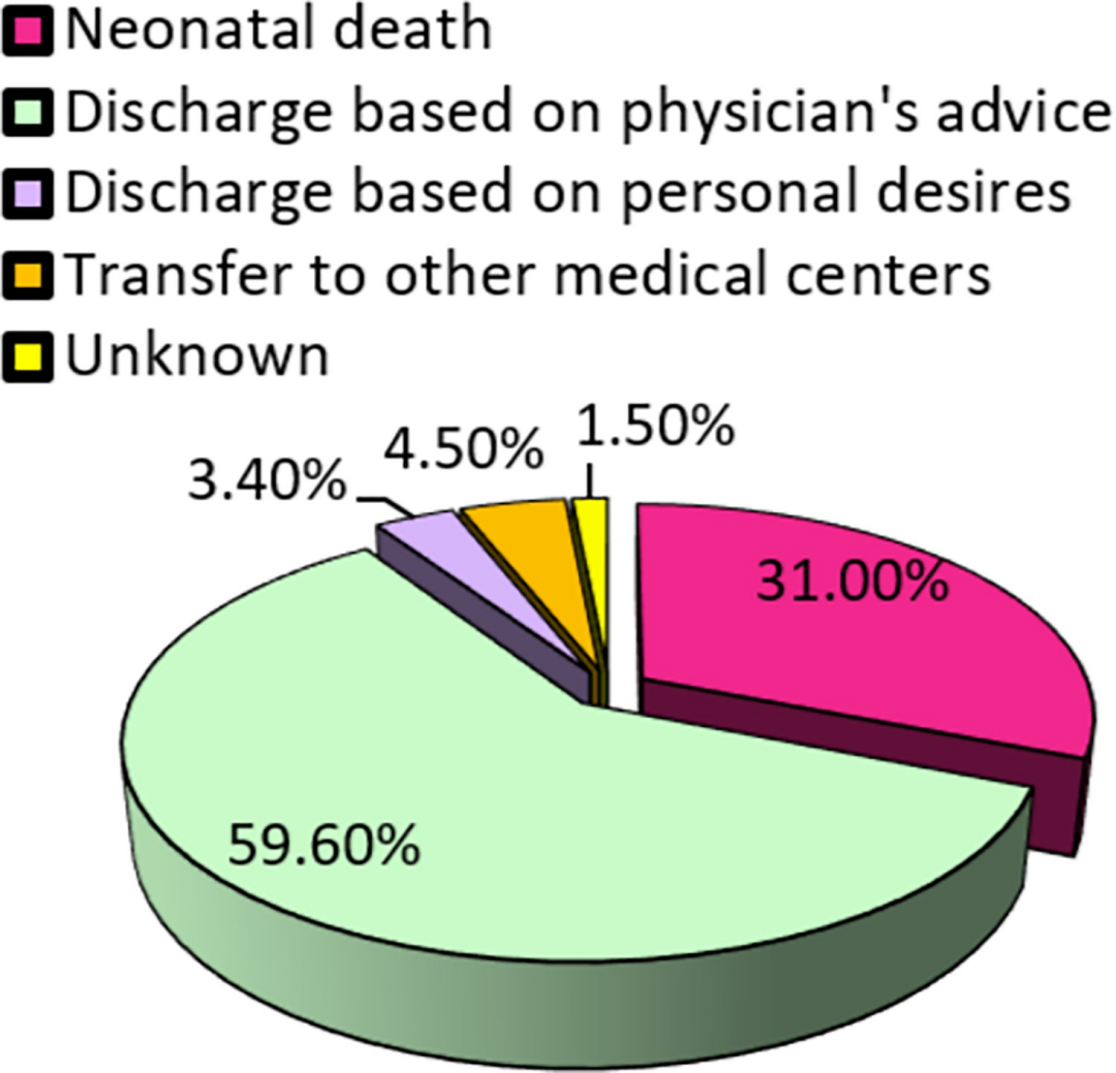
Neonates by fate.

The results show that 62.50% of the neonates had RDS (Fig 1). It was also found that 59.60% of the neonates were discharged from the hospital at a doctor’s discretion, but 31% of them died (Fig 2)

### 3.1 Drug selection index (Combination of four indicators)

The drug selection index showed that 14.3% of these prescriptions did not comply with any of the rational prescription indicators; in fact, these neonates did not need to receive surfactant at all. The results of this index were surprisingly similar in terms of the national and international guidelines (Tables 1 and 2).

**Table 1:** Index of drug selection based on instrument 1) national guideline).

Prescription indicators	Indicator variables	Different situations resulted from reviewing prescriptions		
		Rational	Irrational	Unverifiable
Indicator 1: Variables a and b	Variable a: Gestational age	≤ 259 days (37weeks (	>259 days 37)weeks)	Missing data
	Variable b: Advancement in resuscitation operations in the operating / delivery room	In need of resuscitation by intubation in the operating / delivery room	No need to resuscitation by intubation in the operating / delivery room	
	Number	147 (17.37)	688 (81.32)	11 (1.30)
Indicator 2: Variables a and b	Variable a: gestational age	≤ 259 days37) weeks(	> 259 days 37)weeks)	Missing data
	Variable b: Type of Respiratory support before surfactant prescription and Status of the need for increasing CPAP and FIO_2_	NCPAP and need to increase CPAP > 8 cm/H_2_O or FIO_2_ > 30%	No- NCPAP and no need to increase CPAP > 8 cm/H_2_O or FIO_2_> 30%	
	Number	456(53.90)	369(43.61)	21(2.40)

Indicator 3: Variables a and b	Variable a: gestational age	≤ 259 days 37) weeks (	>37) weeks) 259 days	Missing data
	Variable b: chest radiograph during the first 48 hours after birth	Abnormal (with typical RDS radiographic signs)	Normal (without any typical RDS radiographic sign)	
	Number	474(56.02)	363(46.90)	9(1.06)
Indicator 4: Variables a and b	Variable a: Type of the respiratory distress disease	RDS or MAS ^1^ or PNA ^2^	No- RDS & no-MAS & no-PNA	Missing data
	Variable b: Advancement in resuscitation operations	Use of endotracheal intubation	Use of no endotracheal intubation	
	Number	290(34.30)	533(63.00)	23(2.70)
Drug selection index (combination of these 4 indicators)		(83.80) 709^3^	(14.30)121	(1.89)16

^1^ Meconium aspiration syndrome.

^2^ Intrauterine pneumonia.

^3^ Out of a total of 709 neonates with rational index of drug selection, rational surfactant selection index was confirmed for 18 neonates in all these four indicators, for 167 neonates in three indicators, for 258 neonates in two indicators, and for 266 neonates only in one indicator.

**Table 2:** Index of drug selection based on instrument 2) international guideline).

Prescription indicators	Indicator variables	Different situations resulted from reviewing prescriptions		
		Rational	Irrational	Unverifiable
Indicator 1: variables a or b	Variable a: Clinical diagnosis	Diagnosed RDS or Abnormal chest radiograph	No- RDS and Normal chest radiograph	Missing data
	Variable b: Chest radiograph			
	Number	529(62.52)	311(36.76)	6(0.70)
Indicator 2: variables a or b	Variable a: Birth weight	Weight ≤1300 g	Weight >1300 g	Missing data
	Variable b: Gestational age	Age ≤ 224 days (32weeks)	Age>224 days (32weeks)	
	Number	446(52.71)	400(47.28)	0(0)
Indicator 3: Variables a and b	Variable a: intubation	Intubation	Other clinical conditions	Missing data
	Variable b: Fio_2_^1^	Fio2 ≥40		
	Number	414(48.93)	399(47.16)	33(3.90)
Drug selection index (combination of 3 indicators)		719(84.98)	122(14.42)	5(0.59)

^1^ Fraction of inspired oxygen.

### 3.2 Dose index

The findings reported in Table 3 show Curosurf surfactant as the most common and Alveofact as the least commonly used surfactant prescribed for neonates.

**Table 3:** Dose index and frequency of overdose and under-dose by surfactant type.

Type of Surfactant	Total Prescriptions	Prescriptions with unverifiable Dose	Prescriptions with rational dose		Prescriptions with irrational dose		Irrational dose mean ± SD	
			Rational	Rational without sensitivity	Over dose	Under dose	Over dose	Under dose
Curosurf	487(57.97)	132(53.22)	213(88.75)	18(15.00)	97(64.66)	27(32.92)	1.12±1.77	0.50±0.45
Survanta	239(28.45)	88(35.48)	14(5.83)	57(47.50)	33(22.00)	47(57.31)	3.42±3.81	2.00±2.12
BLES	84(10.00)	20(8.06)	10(4.16)	29(24.16)	19(12.66)	6(7.31)	3.61±5.55	2.03±1.99
Alveofact	30(3.57)	8(3.22)	3(1.25)	16(13.33)	1(0.66)	2(2.43)	0.30±1.93	0.78±0.21
Total^1^	840(100)	248(100)	240(100)	120(100)	150(100)	82(100)	2.27±3.10	1.12±1.34
Dose index	846(100)^2^^, 3^	248(29.31)	360(42.55)		232(27.42)		1.69 ±2.22	

Data are presented as mean ± standard deviation and number (%).

^1^Row 5: Vertical sum of percentages.

^2^ Horizontal sum of percentages related to dose index.^3^ The surfactant type was unknown in 6 cases (0.70).

### 3.3 Time index

The time index indicated wrong-time prescriptions in only about 1% of the cases (Table 4).

**Table 4:** Prescription time index.

Prescription time	Description	Time index
Rational	The first 48 hours after birth	804(95.03)
Irrational	After the first 48 hours after birth	9(1.06)
Unverifiable	Missing data	33(3.90)

Data are presented as number (%).

### 3.4 Total Index of Drug Prescribing (TIDP)

The total drug prescription index showed that 32.86% (n = 278) of the prescriptions were rational (TIRP), i.e. these patients received the right drug at the right dose and right time. As well, 37.47% (n = 317) of the prescriptions were irrational (TIIP), of which 188 patients had a rational drug selection index, but they received an irrational dose. Of note, there were only two patients with rational drug selection index and dose index; however, they received the drug at the wrong time. Finally, 29.66% (n = 251) of the prescriptions were unverifiable, of which more than 92% were unverifiable due to missing dose index data. (Tables 5–7).

**Table 5:** Combination of drug selection, and dose indexes.

Drug selection index	Dose index	Different situations resulting from the combination of two indexes	Combination of drug selection-dose indexes
Rational	Rational	Rational	288(34.04)
	Irrational	Irrational	188(22.22)
	Unverifiable	Unverifiable	233(27.54)
Irrational	Rational	Irrational	66 (7.80)
	Irrational	Irrational	41(4.84)
	Unverifiable	Irrational	14 (1.65)
Unverifiable	Rational	Unverifiable	6 (0.70)
	Irrational	Irrational	3 (0.35)
	Unverifiable	Unverifiable	7 (0.82)

**Table 6:** Total index of drug prescribing (Combination of the drug selection, dose, and time indexes).

Combination of drug selection-dose indexes	Time index	Total index of drug prescribing	
		Different situations resulted from the combination of these three indexes	Number(%)
Rational	Rational	Rational	278 (32.86)
	Irrational	Irrational	2 (0.23)
	Unverifiable	Unverifiable	8 (0.94)
Irrational	Rational	Irrational	301 (35.57)
	Irrational	Irrational	4 (0.47)
	Unverifiable	Irrational	7 (0.82)
Unverifiable	Rational	Unverifiable	225 (26.59)
	Irrational	Irrational	3 (0.35)
	Unverifiable	Unverifiable	18 (2.12)

Data are presented as number (%).

**Table 7:** Status of total prescription index and its variables.

Evaluation status	Instrument 2	Instrument 1			
	Drug selection index	Drug selection index	dose index	Time index	Total index of drug prescribing
Rational	(84.98)719	709 (83.80)	360 (42.55)	804 (95.03)	278 (32.86)
Irrational	(14.42)122	121 (14.30)	232 (27.42)	9 (1.06)	317 (37.47)
Unverifiable	(0.59)5	16 (1.89)	254 (30.02)	33 (3.90)	251 (29.66)

Data are presented as number (%).

### 3.5 Prescription habits

The prescription time index with 95% rational prescription was the first and the most frequent appropriate prescription habit. The indicators 3 and 2 accounted for 56.02% and 53.90% of rational prescriptions; thus, they were the second and third appropriate prescription habits, respectively. Dose index accounted for 27.40% of irrational prescriptions and was recognized as the only inappropriate prescription habit in this regard.

## 4 Discussion

The present study aimed to determine the rate of irrational surfactant prescriptions among neonates with respiratory distress disease. Overall, the results show that in Iran, 37.47% of all the surfactant prescriptions (53.27% of verifiable prescriptions) were irrational in neonates.

### 4.1 Neonates’ real need for surfactant

The results of the investigation of drug selection index using two national and international instruments were surprisingly similar, both of which showed that this index was irrational for about 14% of neonates, i.e. these neonates did not need to receive surfactant at all.

Although drug selection error may be conscious, economists and policymakers for a long time have been arguing that health care providers demand health services from society by using information asymmetries and providing services whose values are questionable [21, 22]. On the other hand, this error type may be completely unintentional and caused by poor medical knowledge, so it may not be provided continuously in-service training to physicians, which leads them to rely on prescription methods they have learned from their studies [23, 24]. Furthermore, inadequate education is known as another reason for poor medical knowledge among physicians leading to misdiagnosis [25, 26]. In consistent with the results of the present study, Amman et al. (2018) in their study have investigated 2682 prescribed drugs and found that 58.50% of these prescribed drugs were potentially inappropriate [27]. In another similar study, Aucejo et al. (2019) have investigated 16562 prescriptions in a French children’s hospital. As a result, they found that respiratory diseases were the most commonly prescribed and potentially inappropriate drugs [28].

To solve such problems, evidence has shown that Educational interventions can, to a large extent, improve and change physicians’ prescribing behaviors [29]. Another solution to reduce irrational prescribing of this index is the use of clinical decision support systems (CDSS). Accordingly, this technology links clinical evidence with medical knowledge [30]. In addition, in Iranian teaching hospitals, the residents of the third and fourth years of pediatrics are currently allowed to prescribe surfactant with the approval of the head of the department, so it may be necessary to take stricter measures in determining eligible individuals to prescribe surfactants for neonates.

### 4.2 Deviation of the prescribed dose from the standard dose

The results of this study show that 39.18% of the total evaluated doses were irrational, and of the total of irrational doses, 64.65% were overdose and 35.34% were under-dose. In this regard, other researchers have also stated that the absence of medical guidelines, lack of rules for prescribing and non-monitoring of prescriptions are the possible reasons for various types of medication errors in this field [16]. It has been cleared that neonatal medication errors are often caused by dose miscalculation. Moreover, one of the causes of dose errors is miscalculation of the newborn’ weight, which consequently leads to miscalculation of the weight-based dose [31].

In this regard, Iftikhar et al. (2019) in their study have investigated the errors of prescribing antibiotics for neonates with respiratory infections. They showed that 40.80% of the prescriptions were wrong, of which 19.90% were due to dose error (13.70% and 6.30% belonged to under-dose and overdose, respectively) [32]. In another study, Jones et al. (2017) have determined the prevalence of rounded drug doses (0.01 for NICU and 0.10 for not-NICU). Furthermore, they showed that the prevalence rate of this error in NICU is 55.90% and NICU children are at higher risk compared to not-NICU children. Overall, 71% of doses were rounded [33].

To reduce dose error, training seems to be necessary to strengthen the skill related to calculating the weight-based dose. Moreover, this type of drug error is reduced using computerized physician order entry (CPOE), due to the reason that the weight-based dose is calculated automatically [33]. In this regard, using weight-based dose charts by type of surfactant is known as a simple solution, which leads to the increased speed, accuracy, and patient’s safety [34]. Besides, the application of the application of the "sterile cockpit rule” can be helpful as well. This strategy was firstly introduced in the aviation industry in the 1980s to prevent unnecessary activities and conversations at critical flight stages in the cockpit [35]. Currently, many health centers use this rule to eliminate distractors while providing services. According to previous studies, this rule leads to a reduction of 42.78% in medication errors [36].

### 4.3 Surfactant replacement therapy at the right time

The time index was found to be rational in 95% of prescriptions. In consistent with the results of the present study, a previous study also confirmed that preterm neonates often receive surfactant either during the first 24 hours of life (day 0) or in the second 24 hours of their life (day 1) [37]. In line with the present study, another study reported that only 4.50% of all NICU prescribing errors are related to time [38], but Singh (2019) has shown that the treatment time was the most common medication error with a 27.50% fault rate [39]. It is worth noting that comparing the results of one study with those of other studies is not always appropriate due to the use of different definitions of rational prescription and different methods of analysis. To reduce time error, we suggest that hospital officials should identify a list of time-critical medicines and include training programs on prescription time of these medications in hospital policies [40].

### 4.4 Choosing the right drug at the right dose at the right time (TIDP)

In this study, the findings related to the total prescription index showed that out of 846 reviewed medical records, 251 were unverifiable (IUNVERP), and the main reason was the dose-related missing data on the doctor’s prescription sheet. Other studies have also confirmed that parts of a patient’s medical record that must be completed by a physician have more missing data compared to other parts [41]. Since health records are the important sources of protection for the legal rights of patients, physicians, and health care centers [42], it is essential that all individuals who document patient’s information, should be aware of its importance and know about the governing rules of health records [43].

As well, in the present study, the total drug prescription index showed that 278(46.72% of IVERFP) were rational prescriptions and 317 were irrational prescriptions (53.17% of IVERFP). In other words, 278 neonates received the right drug at the right dose and right time. Of 317 neonates who were prescribed irrational doses, 121 neonates had wrong surfactant selection, and irrational prescription was observed in 191 neonates due to wrong dose as well as 5 neonates due to wrong time.

Machado et al. (2015) have conducted a study to measure the rate of prescription error according to the NeoFax prescribing protocol in the NICU. As a result, they reported the rate of prescription error as 43.50% and the fourth highest error rate was found to be related to the respiratory drugs [44]. In another similar study, Campino et al. (2009) have shown that the prescription error rate is 20.70% in the NICU [45].

One of the main limitations of the present study was that, according to national standards, we expected medical records to be electronically available in hospitals. However, this was not the case in some of the hospitals, and the research team had to wait for the medical records to be scanned at first and then sent; therefore, the study period was longer than what was anticipated.

## 5 Conclusion

The present study aimed to determine the rate of irrational prescription in neonatal surfactant therapy. In this research, among the known three components of the total drug prescription index, time index with the least error was found as the most frequent appropriate prescription habit and dose index with the most error was identified as the only inappropriate prescription habit. Finally, it was found that only 32.86% of neonates received the right drug at the right dose and right time. Considering that at least one university of medical sciences exists in each province of Iran and due to the reason that these universities are responsible for managing, policy-making, and providing services in their affiliated hospitals, it is necessary to play more active roles in monitoring the rational use of surfactant and using practical interventions in this regard.

## Supporting information

S1 Data (SAV)
